# Friend or Foe? Biomechanics and Its Key Role in Paediatric Orthopaedics

**DOI:** 10.3390/children11010090

**Published:** 2024-01-11

**Authors:** Jaap J. Tolk, Pieter Bas De Witte

**Affiliations:** 1 Department of Orthopaedic Surgery and Sports Medicine, Sophia Children’s Hospital, Erasmus University Medical Center, 3000 CA Rotterdam, The Netherlands; 2 Department of Orthopaedics, Leiden University Medical Center, 2333 ZA Leiden, The Netherlands; p.b.de_witte@lumc.nl

## 1. Introduction

Biomechanics play a key role in the development, progression and treatment of musculoskeletal disease in children. These biomechanics can be either friend or foe. On the one hand, there is excellent remodeling potential; on the other hand, as just one example, growth plate injuries and consequent growth disturbances can lead to significant problems. 

There is a complex interaction between mechanical factors, growth and development, movement and physical function. Understanding these biomechanical factors is crucial in optimizing diagnostic and treatment strategies and improving outcomes for children with orthopaedic conditions.

In this Special Issue of *Children*, we address biomechanics in paediatric orthopaedics. A wide range of quality papers regarding this subject are included, increasing our knowledge and understanding of the complex relationship between pathology, skeletal growth and mechanical factors during childhood.

## 2. Biomechanics as a Friend

On the one hand, biomechanical aspects of the growing skeleton can often be used as an advantage during treatment, for example, in clubfoot correction with stepwise casting and/or tendon transfers or the treatment of limb length and/or axis discrepancies. 

A strong example of the beneficial use of growth and biomechanics in the treatment of children with orthopaedic conditions is the application of guided growth techniques. These techniques involve surgical procedures around the growth plates, for example, by drilling (epiphysiodesis) or with the use of temporary implants, such as tension plates, screws or staples, to modulate the growth of a specific bone or limb. These implants alter the mechanical forces acting on the growth plate, allowing for the correction of angular deformities (hemi-epiphysiodesis) or limb length discrepancies. 

The above methods are well known and widely applied for leg length differences or leg varus/valgus correction procedures around the knee [1,2,3]. However, several papers in this Special Issue discuss novel applications of guided growth techniques.

Lebe et al. show that the guided growth of the proximal femur has great potential as a low-invasive treatment method for hips at risk of dislocation in cerebral palsy patients. They conclude that the technique is effective and predictable with an overall low complication rate; however, further work is required to identify the best candidates and surgical timing, as well as choice of technique and implant [Contribution 1].

Paley and Shannon explore the application of guided growth beyond angular and longitudinal deformities, focusing on the correction of rotational deformities in the lower extremities using a flexible tether device. In their preliminary study, they concluded that rotational guided growth can successfully correct torsional malalignment without invasive osteotomy surgery [Contribution 2]. 

Though less invasive than corrective osteotomies, guided growth techniques are not without morbidity. Braun et al. show that in the treatment of valgus deformities with hemi-epiphysiodesis, a relevant number of patients sustain prolonged pain and limited mobility. Patients with simultaneous plate implantation at the femur and tibia, as well as metaphyseal plate positioning, experienced resulting prolonged pain and a delay in functional recovery. These findings highlight the importance of carefully considering the specific indications and techniques used in guided growth procedures to minimize complications and optimize patient outcomes [Contribution 3].

Additionally, for guided growth, biomechanics can be a friend in other ways. For example, Segal et al. analysed a cohort of patients with cerebral palsy and assessed the potential beneficial effect of using a Functional electrical stimulation of the ankle dorsiflexor (DF-FES). They found limited benefit of the DF-FES on gait parameters; postural control seemed to be improved at the cost of a slower but more controlled gait [Contribution 4]. Furthermore, Gangaram and colleagues studied pre-operative traction before closed reduction in children with developmental dysplasia with a dislocated hip. In a retrospective pair-matched study, they analysed whether maintenance of hip reduction was influenced by the application of pre-operative longitudinal traction. They conclude that traction treatment does not significantly improve the short-term or mid-term outcomes for closed reduction and, therefore, should not be used as standard care for dislocated hips in Developmental Dysplasia of the Hip (DDH) [Contribution 5].

## 3. Biomechanics as a Foe

Disadvantageous effects of biomechanics in the growing skeleton have to be considered as well; examples include physeal injuries leading to growth disturbance or the progression of idiopathic scoliosis during adolescence [3,4]. 

In a large longitudinal cohort of hip dysplasia patients, Merchant et al. show that leg length differences in children treated for DDH are common. In two-thirds of the patients, the affected DDH leg was longer, mainly arising from the subtrochanteric segment. On the other hand, patients with a higher grade of AVN were often found to have shortening of the DDH leg. Interventions to correct leg-length differences were performed in 27.5% of their patients. They recommend careful monitoring of LLD in the follow-up of patients with DDH [Contribution 6].

Jansen et al. reviewed windswept deformities in children. These deformities can be a tell-tale sign of underlying disorders and can significantly impact daily functioning. The authors present a literature overview with a step-by-step guide for clinicians who encounter a child with windswept deformity [Contribution 7].

## 4. The Role of Biomechanics in Research and Diagnostics

Lastly, biomechanics can be utilized in the analysis of the consequences of structural skeletal abnormalities on joint mechanics and function for research or diagnostic purposes or for the development of innovative surgical techniques [5].

The influence of structural orthopaedic disorders on gait and physical function is an example of the strong interaction with biomechanical factors. A series of papers assessing this interaction, using instrumented gait analysis, could be included in this Special Issue.

Liu et al. summarize the available literature on gait characteristics in typically developing toddlers, providing a reference for clinical assessment and further clinical research [Contribution 8]. And in a systematic literature review, Grin et al. provide an in-depth analysis of the range of kinematic gait differences that can be expected in children with clubfoot treated with the Ponseti method, as compared to healthy controls. They provide strong recommendations for future research, with the implementation of multi-segmental foot models and a focus on the relationship between gait impairments and functional problems [Contribution 9].

## 5. Conclusions

This Special Issue provides a comprehensive overview of advancements in orthopaedics and biomechanics in children, highlighting topics such as guided growth, structural skeletal alignment and length and the impact of orthopaedic disorders on gait and physical function. The studies described above, combined with the contributions of Moerman et al., Hollyer et al. and Fernandez-Perze et al. [Contributions 10–12], suggest that future research should concentrate on analyzing the application of guided growth beyond longitudinal and angular abnormalities, with specific attention to appropriate patient selection and the assessment of long-term outcomes. Additionally, further investigation into the relationship between gait deviation, physical function and the impact of treatment modalities is needed to enhance our understanding in this area. Research papers presented in this Special Issue add to the scientific basis for further research and the improvement of patient care for children with orthopaedic disorders in the future.
